# Measuring autonomy in hybrid work: scale development

**DOI:** 10.1186/s41155-025-00338-1

**Published:** 2025-03-28

**Authors:** Pallavi Datta, Sathiyaseelan Balasundaram, Elangovan N, Sridevi Nair

**Affiliations:** School of Business and Management, Christ University, Bengaluru, Karnataka 560029 India

**Keywords:** Autonomy in work, Hybrid workplace, Scale development, Information Technology Industry, India

## Abstract

**Background:**

Autonomy is a core element in many established management theories, consistently linked to positive employee outcomes. However, the COVID-19 pandemic and rapid technological advancements have transformed workplace dynamics, particularly in the information technology (IT) sector in India, where hybrid work models have gained prominence. Despite this shift, no standardized measure exists to assess the autonomy experienced by employees in hybrid work environments, hindering deeper analysis and understanding.

**Objective:**

This study aims to contextualize, develop, and validate the Autonomy in Hybrid Work Scale (AHWS) for the Indian context, providing a tool for researchers and practitioners to systematically examine the impact of autonomy in hybrid work.

**Methods:**

A descriptive two-phase study was conducted following DeVellis’s scale development framework. Phase 1 focused on conceptualizing and developing the construct through a comprehensive literature review, item generation, and assessment of content and face validity by experts, followed by a pilot test. Phase 2 encompassed the scale validation process, which included Exploratory Factor Analysis (EFA) to identify the underlying factor structure and Confirmatory Factor Analysis (CFA) to validate the model and assess its fit.

**Results:**

The data collected from 313 IT employees working in Bengaluru, India, was analyzed to confirm data normality (below ± 2.58). The items showed a strong and positive correlation (*r* = .734) with the Work Design Questionnaire which indicated convergent validity. Discriminant validity was confirmed through Fornell-Larcker and Heterotrait-Monotrait (HTMT) criteria, with HTMT values below 0.90. The final analysis yielded an 18-item scale with a Cronbach’s alpha of 0.825, comprising four distinct dimensions: (a) work location autonomy, (b) work time autonomy, (c) work scheduling autonomy, and (d) work decision autonomy.

**Implications:**

The AHWS offers a valuable tool for both managers and academics to assess how different forms of autonomy influence employee well-being and productivity in hybrid work settings. It also addresses a gap in the literature, providing a foundation for further empirical research on autonomy in hybrid work models.

## Introduction

Spiegelaere et al. (2016) have defined Job autonomy as “the discretion of employees to complete tasks when, where, in what order and in what way.” It is also referred to as “work autonomy” or “job control,” and it plays a significant role in influencing employee outcomes. The concept of work autonomy has constituted a subject of significant scholarly attention within management studies and social sciences over several decades (Campion, 1988; Hackman & Oldham, 1975; Korunka & Kubicek, 2017). Furthermore, the COVID-19 pandemic and the rapid evolution of technology have induced a transformative shift in the operational landscape of work, thereby mandating a comprehensive reappraisal of the definition and measurement of work autonomy within the contemporary milieu (Miller, 2022). With the rise of the hybrid workplace, where employees work remotely and in the office, work autonomy has taken on a novel dimension, requiring employees to be more self-directed and take charge of their work to meet the demands of the virtual environment (Yang et al., 2021).

A recent investigation in the Harvard Business Review aimed to analyze the future of work arrangements. The analysis of selected 5000 knowledge workers worldwide showed the following results: 77% said they would prefer to work for a company that allows them to function from anywhere rather than a fancy corporate headquarters. However, with 61% of knowledge workers reporting that they would favor it if management permitted them to come into the office when they need to, their data indicated that the flexibility they seek is contingent on their ability to utilize it in a form that accurately suits them. In summary, the reliance on flexibility depends on their “autonomy” (Reisinger & Fetterer, 2021). Taking into consideration that these words possess different interpretations holds substantial significance.

Commonly, workplace flexibility is defined as the opportunity to adjust the when, where, and how of work (Cowan & Hoffman, 2007). Nevertheless, in practicality, the flexible schedule is commonly pre-determined by the management or the employer (Bloom, 2021). For instance, employees can work from home on Mondays and Wednesdays every week, or employees can work remotely only 2 days a week. However, in the present context, when employees refer to the term “flexibility,” they represent the concept of “autonomy.” Consequently, the data shapes a vision of a future work landscape centered on autonomy-driven flexibility. It indicates that hybrid work strategies that solely focus on flexibility without autonomy are likely to be substandard or rejected by most employees (Forum, 2022; Reisinger & Fetterer, 2021; Reporting, 2022).

While a substantial number of studies has lauded the favorable outcomes of work autonomy on employee wellbeing and motivation (Karasek, 1979), some empirical evidence suggests that excessive levels of job autonomy, after a certain point, may no longer affect employees’ mental health or even be detrimental (Warr, 2009). Accordingly, it is crucial to understand whether a certain extent of autonomy greatly benefits employee outcomes and whether it is worth it for the management to make amendments to workplace policies (Datta et al., 2023). Identifying its ramifications on employee outcomes would enable organizations to design a suitable hybrid work model that fosters a healthy balance between autonomy and control and promotes positive outcomes for employees and management (Allvin et al., 2011; Flecker et al., 2017).

## Research problem

Despite the growing adoption of hybrid work arrangements, there is currently no established tool to quantify the degree of autonomy endowed to the employees. Existing scales either lack precision or have a different focus (Prem et al., 2020). For instance, the Work Design Questionnaire, as developed by Morgeson and Humphrey (2006), assesses initiated and received interdependence but does not account for autonomy in coordinating with others. Likewise, established scales pertaining to flexible work, such as those by Shockley and Allen (2007), primarily concentrate on the availability or utilization of temporal and spatial flexibility. These measurement tools do not encompass the extent of autonomy concerning the planning of work-time and workplace, as pointed out by Allvin et al. (2011) and Prem et al. (2020). While some existing scales address flexible or deregulated work, they often have different focal points and typically encompass other aspects of evolving work conditions, such as requirements for self-directed career advancement, learning, and effort management (Prem et al., 2020). The dearth of a measurement tool for assessing autonomy in a hybrid workplace could drive forthcoming studies to yield indefinite and contrasting employee outputs. Developing an accurate scale to eliminate these distinctive results and enable researchers and practitioners to examine autonomy in hybrid work with reliability is crucial (Datta et al., 2023). Considering the preceding discussion, the objective of the current study is to:

- Define the construct of “autonomy in hybrid work”
- Develop and validate a multi-dimensional scale
- Suggest the implications of the newly developed scale

## Review of literature

### The concept of autonomy in work

Work autonomy, often referred to as “job autonomy” or “job control,” plays a significant role in influencing employee outcomes. Table 1 provides an overview of the existing definitions and dimensions of autonomy in work. The information provided in Tables 1 and 2 is obtained through a keyword search in Google Scholar, Scopus, and Web of Science database of words; “autonomy,” “job autonomy,” “work autonomy,” “scale development,” “scale validation,” “measuring autonomy,” “dimensions of autonomy,” and “autonomy definition” in different combinations.

**Table 1:** Evolution of definitions and dimensions of autonomy in work

Source	Definition	Dimension
(Hackman & Oldham, 1975)	“The degree to which the job provides substantial freedom, independence, and discretion to the individual in scheduling the work and in determining the procedures to be used in carrying it out.”	1. Work procedure 2. Work schedule
(Breaugh, 1985)	“Work autonomy may be defined as the degree to which an individual is given freedom and discretion in carrying out a task.”	1. Work method autonomy 2. Work schedule autonomy 3. Work criteria autonomy
(Morgeson & Humphrey, 2006)	“Autonomy reflects the extent to which a job allows freedom, independence, and discretion to schedule work, make decisions, and choose the methods used to perform tasks.”	1. Work scheduling autonomy 2. Decision making autonomy 3. Work method autonomy
(Spiegelaere, et al., 2016)	“Job autonomy refers to the discretion of employees to complete tasks when, where, in what order and in what way.”	1. Work method 2. Work scheduling 3. Work time 4. Place of work

Source: Accumulated by authors

**Table 2:** Existing scales to measure work autonomy in a traditional workplace

Source	Scale name	No. of items	Country
Breaugh (1985)	Work Autonomy Scale	9 items	USA
Jackson et al. (1993)	New Measures of Job Control	10 items	UK
De Jonge (1995)	Maastricht Autonomy Questionnaire (MAQ)	10 items	Netherlands
Spector and Fox (2003)	Factual Autonomy Scale (FAS)	110 items	USA
Morgeson and Humphrey (2006)	Work Design Questionnaire (WDQ)	9 items	USA

Source: Accumulated by authors

Many of the conceptualizations of work or job autonomy were formulated in the 1980s or 1990s, and as a result, they may not adequately address the contemporary shifts in the way of working. The recent literature review does not address the evolving changes in the concept, and hence, many older articles were referred to understand the construct in-depth. While the dimensions of job autonomy mentioned earlier primarily pertain to the job’s task level, the latest developments, such as the proliferation of flexible work arrangements, have introduced autonomy at the job level (Korunka & Kubicek, 2017). Nowadays, employees also have the autonomy to decide where and when they carry out their job duties (Gerdenitsch, 2017).

### Existing scales of autonomy in work

Measurement is critical across various social research contexts (DeVellis, 2021). They are essential for assessing various phenomena in research and real-world settings (Linden & Hambleton, 1996). Besides, it is significant to note that specific measurement scales suit specific contexts (Stevens, 1946). Therefore, it is essential to utilize a dedicated measurement tool for assessing distinct workplaces, as the unique characteristics of each workplace demand tailored evaluation methods (Fried & Ferris, 1987; Shoss et al., 2013). Multiple scholars have introduced diverse scales for assessing autonomy in traditional workplaces. The notable measurement instruments utilized to measure autonomy in work have been traced from the existing literature and are illustrated in Table 2.

Table 2 demonstrates the consistent emphasis on autonomy in work methods and scheduling by researchers over the years (Breaugh, 1985; Hackman & Oldham, 1974). Notably, the most widely cited autonomy scale, with over 3640 citations on Google Scholar, was established in 2006 (Morgeson & Humphrey, 2006), marking eighteen years since its inception. None of the autonomy measurement scales mentioned in Table 2 addresses the hybrid work context and neither has been designed for the Indian population.

A McKinsey report from August 2023 predicted India’s growing working-age population by 2030 (Kandasi, 2023). Furthermore, most Indian employees see hybrid working as a sustainable option (Sarkar, 2023), and 73% of companies in India are considering its adoption (Gautam, 2022). Leading global organizations like Intel, JP Morgan, Cisco Systems, Oracle, American Express, Accenture, and Adobe Systems have already adopted the hybrid work model in India (Gupta, 2023). This trend underscores the importance of developing a tailored autonomy scale for India’s unique workplace characteristics and practices.

### Defining hybrid work

The term “hybrid work” gained attention during the COVID-19 pandemic in India (Hopkins & Bardoel, 2023). It emerged due to factors like long commutes, cost pressures on office spaces, hotdesking, and changing architectural trends (Halford, 2005). “Hybrid” is now a broad label encompassing various work-related concepts like hybrid workplaces, work, and teams (Appel-Meulenbroek et al., 2022; Fayard et al., 2021; Hatfield & Pearce, 2022; Keane & Heiser, 2021; Knight, 2020; Smet et al., 2021).

Different authors define hybrid work in diverse ways. Some focus on location flexibility (Halford, 2005; Moglia et al., 2021), while others include time flexibility (Gratton, 2021; Smite et al., 2023). Although a universal definition is lacking, it emphasizes employee flexibility (King’s College London, 2021).

For this study, the definition by Hopkins and Bardoel (2023) is adopted: “a work arrangement where employees divide their time between a traditional workplace and remote locations, like their homes or ‘third places’ such as coworking spaces, libraries, or cafes.”

## Research design

A descriptive study was undertaken due to its suitability for capturing and presenting numerical data elucidating the phenomenon’s characteristics under investigation. The methodological aspect of the study is cross-sectional. In this study, the “Autonomy in Hybrid Work Scale” (AHWS) was developed following the DeVellis Scale Development process (DeVellis, 2016) and validated stepwise. The procedure stated in the book, “Scale Development: Theory and Applications” has more than 36,000 citations in Google Scholar, and many authors have developed and published new scales in Q1 Scopus journals like the International Journal of Human Computer Studies (Salminen et al., 2020) and Management Communication Quarterly (Fuller et al., 2019) among others. The process produces a comprehensive and systematic approach to scale development, encompassing various stages from item generation to psychometric evaluation. The scale was developed and validated in two phases conducted during different time frames to ensure the instrument accurately captured the constructs under investigation.

### Sampling design and data collection

IT knowledge workers contend with challenges like long hours, demanding schedules, competition, and extended VDU exposure, causing occupational stress and health risks. This yields issues such as psychological distress, reduced commitment, anxiety, job dissatisfaction, absenteeism, and high turnover rates, a concern for senior management. IT organizations and HR practitioners must devise diverse strategies, emphasizing innovative solutions to assist employees in overcoming these challenges (Malik & Garg, 2017). The study focused on IT professionals in Bengaluru, India, known as the IT capital of India, with a significant concentration of over 67,000 registered IT companies and 75% of IT professionals. More than 80% of Indian IT organizations are inclined to adopt a hybrid work model (Baruah, 2022). Creating a scale to assess autonomy in hybrid work is crucial for effective management in such a stressful work environment.

According to Hair et al. (2014), a general rule of thumb is that 10 participants are required for each item to conduct Exploratory Factor Analysis (EFA). Since this study included 18 items, a minimum of 180 responses was necessary for EFA. Additionally, Kline (2015) recommends a minimum sample size of 200 for conducting Confirmatory Factor Analysis (CFA). Therefore, to meet the higher threshold, the minimum sample size for data analysis in this study was set at 200.

With a survey response rate of approximately 57% for IT companies in India (Krishnan & Poulose, 2016), the study distributed 120 questionnaires in phase 1 and 750 in phase 2, aiming for 50 and 250 valid responses, respectively. Data collection occurred between February to April 2023 with 53 valid responses in phase 1 and May to August 2023 with 349 valid responses in phase 2, resulting in response rates of 44.1% and 46.5%, respectively.

Non-probability purposive sampling, aligned with research objectives, enhanced data, and outcome credibility. Eligible respondents possessed a minimum of 1 year of experience in a hybrid work model and relevant knowledge. Inclusivity of a diverse array of IT companies aimed to encompass different autonomy dimensions offered by various Indian IT organizations (Campbell et al., 2020).

The first phase (scale development) consisted of the following steps: (1) conceptualizing the construct, (2) item generation, (3) content validity, (4) face validity, and (5) pilot study by measuring the discriminant validity, construct validity, reliability, and normality of the responses to the initially developed questionnaire (Devellis, 2016). The five steps outlined above resulted in 24 items, which were subsequently tested for scale evaluation in phase 2 using a sample of 313 respondents.

## Data analysis

Data were analyzed using IBM SPSS Statistics (Version 25) and AMOS (Version 25). Twenty-two forms were excluded from the data collected because they were partly filled and were considered missing data. Further, while analyzing the scores of the social desirability scale by Marlowe and Crowne (2006) on the remaining 327 responses it was found that 14 responses scored 9 out of 13 suggesting that the responses are more likely to reflect socially desirable rather than reality. The 14 responses were removed to avoid the bias of social desirability (Devellis, 2016). Therefore, analysis for phase 2 was performed in the sample size of 313 responses. All data in this study does not contain any missing data.

## Results

The demographic details of the 313 respondents are presented in Table 3. The findings concerning the psychometric properties of the AHWS were presented in two major sections, focusing on validity and reliability.

**Table 3:** Demographic characteristics of the phase 2 sample

Characteristic	Particulars	Frequency	Percent
Gender	Male	123	39.3
	Female	187	59.7
	Other	3	1.0
	Total	313	100
Age	14–25 years	56	17.9
	26–40 years	163	52.1
	41–60 years	78	24.9
	61 and above	16	5.1
	Total	313	100
Work experience in hybrid model	Less than 6 months	20	6.4
	6 months–almost 1 year	63	20.1
	1–2 years	150	47.9
	More than 2 years	80	25.6
	Total	313	100
Current job level	Entry/junior level	143	45.7
	Middle level	130	41.5
	Senior/managerial level	40	12.8
	Total	313	100

Source**:** Author analyzed data

### Validity of the AHWS

Data normality was assessed using mean, standard deviation, variance, skewness, and kurtosis. Mean values (ranging from 2.76 to 3.08) indicate consistent responses (Ghasemi & Zahediasl, 2012). Standard deviation and variance values suggest closely clustered responses. Skewness falls within the acceptable range (± 1), and kurtosis indicates a light-tailed distribution (Jatau Abubakar et al., 2020). With a sample size of over 200, kurtosis values (below ± 2.58) confirm data normality. Next, the corrected item-total correlation assessed communality, yielding acceptable results (range: 0.30 to 0.536) (Mishra et al., 2019) (Table 4).

**Table 4:** Normality and communality tests

Item no	Mean	Std. dev	Variance	Skewness	Kurtosis	Corrected correlation
1	2.91	1.371	1.879	0.130	− 1.288	0.499
2	2.93	1.428	2.040	0.151	− 1.382	0.465
3	2.87	1.446	2.091	0.127	− 1.439	0.536
4	2.93	1.477	2.181	0.152	− 1.449	0.464
5	2.81	1.450	2.102	0.231	− 1.365	0.372
6	2.76	1.400	1.960	0.277	− 1.269	0.413
7	2.81	1.445	2.087	0.261	− 1.339	0.425
8	2.82	1.474	2.173	0.197	− 1.409	0.407
9	2.93	1.357	1.841	0.082	− 1.270	0.424
10	3.08	1.349	1.821	0.041	− 1.302	0.449
11	3.02	1.296	1.679	− 0.036	− 1.169	0.397
12	2.98	1.381	1.906	− 0.045	− 1.307	0.316
13	3.12	1.368	1.872	− 0.106	− 1.306	0.379
14	3.01	1.370	1.878	− 0.012	− 1.264	0.307
15	2.94	1.392	1.938	0.158	− 1.317	0.392
16	2.90	1.383	1.913	0.141	− 1.284	0.378
17	2.99	1.398	1.955	0.058	− 1.326	0.377
18	2.87	1.364	1.860	0.176	− 1.275	0.428

Source: Author analyzed data

The next step involved conducting CFA to establish the construct validity of the scale. Prior to conducting CFA, EFA was employed to assess whether the dataset was appropriate for CFA. The Kaiser–Meyer–Olkin (KMO) value between 0.8 and 1 indicates that the sampling is adequate. Bartlett’s test has a significant value when correlations between variables are large enough to be used in factor analysis. So, Bartlett’s test is appropriate when the significance value is less than 0.05 (Hair et al., 2014). Here, the KMO index of 0.871 and Bartlett test result of *P* < 0.01 (*n* = 313), indicated the dataset’s suitability for factor analysis.

Construct validity, comprising both convergent and discriminant validity, was evaluated using exploratory factor analysis (EFA) with principal component analysis (PCA) as the extraction method and varimax rotation. Convergent validity was assessed by examining whether items exhibited high loadings on their intended factors, indicating strong correlations among items representing the same construct. Discriminant validity was evaluated by ensuring minimal cross-loadings, confirming that each construct was distinct from others. PCA, within the EFA framework, aids in uncovering the underlying structure of the data by analyzing the total variance and grouping variables into factors, as recommended in scale development studies (Hair et al., 2019; Fabrigar et al., 1999). Varimax rotation was applied to achieve factor simplicity and interpretability, supporting the theoretical distinctiveness of constructs. Here, items with weak factor loadings were considered for removal based on two criteria: (a) retaining components with eigenvalues greater than 1 and (b) maintaining items with factor loadings of 0.50 or higher, deemed practically significant (Hair et al., 2014). In the principal component analysis with varimax orthogonal rotation and a sample size of 313, four distinct dimensions were identified (see Table 5). The use of orthogonal varimax rotation facilitated a clearer interpretation of factors by minimizing the number of variables with high loadings on each factor, thus yielding a simpler, more interpretable factor structure with uncorrelated factors (Hair et al., 2014).

**Table 5:** Rotated component matrix

Items	1	2	3	4
I can decide the number of days to work remotely		0.878		
On remote working days, I can choose to work from anywhere (e.g., co-working spaces, home, cafe, etc.)		0.893		
I can shift my workplace in the middle of a workday (e.g., office to home)		0.911		
I can decide which days to work from home		0.900		
I can choose to work for a certain number of hours in a day			0.886	
I can decide when to start and stop working on a task			0.900	
I can work from the office outside the office hours to complete the tasks			0.884	
I can leave the office early when the tasks are completed			0.907	
I can decide the timing and duration of my breaks	0.854			
I can work on holidays from the office	0.910			
I can adjust my work schedule to accommodate personal obligations	0.822			
I can choose to sequence the assigned tasks in an order of my choice	0.883			
While working remotely, I can schedule meetings with my team/manager when required	0.898			
I can decide the amount of work to be done during a certain period of time	0.860			
I can decide to utilize tools and software applications of my choice, to work on the tasks				0.901
I can decide to use a communication channel of my preference to connect with my colleagues to complete tasks				0.879
I can use my personal judgment to complete a task				0.875
I can choose the deadlines for my task submission				0.855

Source:Author analyzed data

Backed with theoretical explanations and distribution of items as presented in Table 5, the authors identified the four dimensions as (1) work location autonomy, (2) work time autonomy, (3) work schedule autonomy, and (4) work decision autonomy. The finalized scale contained 18 items and 4 subscales according to the results of the factor analysis.

The model fit was evaluated using several indices as recommended by both Kline (2015) and Schumacker et al. (2015). The discrepancy divided by the degree of freedom (CMIN/df) value < 3 indicated that the model was usable. Kline (2015) suggested that a minimum index as the root mean square error of approximation (RMSEA) should report values nearer to zero indicating a good fit. Additionally, the model fit was assessed using the Goodness-of-Fit Index (GFI), Adjusted Goodness of Fit Index (AGFI), Normed Fit Index (NFI), Comparative Fit Index (CFI), and Tucker Lewis index (TLI), where values nearer to one indicate a good fit (Hair et al., 2014). The results from the analysis showed that the model is a good fit (Table 6).

**Table 6:** Model fit of the AHWS-18 (*n* = 313)

**Goodness of fit**	**CMIN/df**	**RMSEA**	**GFI**	**AGFI**	**NFI**	**TLI**	**CFI**
Baseline	≤ 3	≤ 0.09	≥ 0.90	≥ 0.90	≥ 0.90	≥ 0.90	≥ 0.90
Results	1.451	0.038	0.939	0.92	0.959	0.984	0.987

Source: Author analyzed data

Construct validity (refer to Table 7), evaluated via convergent and discriminant validity (Boateng et al., 2018), involved a correlation test between AHWS-18 and the Work Design Questionnaire, measuring autonomy (Morgeson & Humphrey, 2006). A strong and positive correlation (*r* = 0.734) indicated convergent validity (Henseler et al., 2014). Composite reliability (CR) for all dimensions exceeded the recommended threshold (0.7), and average variance extracted (AVE) scores for WLA, WTA, and WDA surpassed 0.5 (Brown, 2015). Discriminant validity was confirmed through Fornell-Larcker and Heterotrait-Monotrait (HTMT) criteria, with HTMT values below 0.90 (Fornell & Larcker, 1981).

**Table 7:** Construct validity measures

Constructs	CR	AVE	Fornell-Larcker Criterion and HTMT			
			WLA	WTA	WSA	WDA
WLA	0.951	0.83	0.911			
WTA	0.948	0.821	0.291 (0.316)	0.904		
WSA	0.846	0.49	0.009 (0.052)	− 0.077 (0.097)	0.872	
WDA	0.935	0.782	0.222 (0.241)	0.159 (0.165)	0.008 (0.045)	0.884

Source: Author analyzed data

HTMT values are displayed in parentheses

Moreover, the study assessed the factor invariance of AHWS-18 to examine if the developed scale is interpreted similarly by respondents of different genders. This involved applying a sequence of hierarchical variance models. Following Caycho-Rodríguez et al.’s (2020) guidance, the initial step measured configural invariance (the reference model), followed by evaluating metric invariance (equality of factor loads). Configural variance determined the extent to which the same factors best represented the data for both groups. A two-group analysis tested the unconstrained model, indicating a strong model fit across both groups, suggesting data invariance from a configurable or structural perspective (Table 8). Subsequently, metric variance assessed the equivalence of the construct via factor loading across groups. Factor loading was constrained across groups, and the change in chi-square from the unconstrained to the constrained model was examined. Non-significant values indicated that the meaning of unobservable variables across groups remained the same (Table 8).

**Table 8:** Invariance model by gender

Models	*χ*^2^	df	CMIN/DF	TLI	CFI	NFI	RMSEA	*p*-value
Configural	330.674	258	2.007	0.980	0.983	0.929	0.030	0.000
Metric	342.048	272	1.258	0.982	0.984	0.927	0.029	0.656
Baseline			≤ 3	≥ 0.90	≥ 0.90	≥ 0.90	≤ 0.09	≤ 0.06

Source: Author analyzed data

#### Reliability of the AHWS-18

In phase 2, the scale’s Cronbach alpha was computed at 0.825, with individual dimensions ranging from 0.907 to 0.937, indicating high reliability (Table 9). To reaffirm reliability, composite values were calculated. Following Hair et al.’s (2014) guidelines, the composite value (CR) is a reliable indicator of construct reliability, with a recommended threshold of 0.7. CR assesses internal consistency among all items within the construct (Fornell & Larcker, 1981). The CR values, illustrated in Table 7, notably exceed the threshold, affirming strong internal consistency in the AHWS-18.

**Table 9:** Reliability statistics

Dimension	Cronbach’s alpha (> 0.7)	No. of items
Location autonomy Time autonomy Scheduling autonomy Decision-making autonomy **AHWS-18**	0.932 0.928 0.937 0.907 **0.825**	4 4 6 4 **18**

Source: Author analyzed data

## Discussion

This study aimed to develop the AHWS-18, a comprehensive scale quantifying autonomy in hybrid work, aligning with Korunka & Kubicek’s, (2017) conceptualization. The scale addresses a crucial need for managerial competence in Indian IT organizations, following DeVellis’s (2021) structured process and instilling confidence in the content and construct (Datta et al., 2024; Weerasekara et al., 2021). Initial scale items were crafted through a literature review and expert interviews.

Location autonomy was the first factor identified in the AHWS. It is defined as the “discretion of employees on where to perform the work tasks” by Spiegelaere et al., (2016). Additionally, time autonomy was the second factor identified through the EFA analysis. In accordance with the theoretical contribution by Spiegelaere et al., (2016) and Korunka & Kubicek’s, (2017), it is defined as “the discretion of employees on when to stop and start working.” While Spiegelaere et al. (2016) measured dimensions like location and time autonomy, their focus was primarily on work-from-home employees. For instance, work time autonomy was assessed using a single question, asking respondents to choose between fixed working hours, choosing working hours within limits, or complete freedom in deciding when to start and stop working. Similarly, locational autonomy was measured with a single question on the degree to which respondents could work from home, rated on a 6-item scale ranging from always to never. In contrast, the AHWS-18 scale is meticulously tailored for the nuances of hybrid work, featuring specific items like “I can decide the number of days to work remotely” and “I can leave the office early when the tasks are completed” and others that capture the intricacies of autonomy in the evolving context. By addressing the distinct challenges and opportunities presented by hybrid work arrangements, the developed scale aims to provide a more targeted and relevant assessment tool for researchers and practitioners navigating the complexities of contemporary work environments. Also, it is important to note that autonomy in terms of time is a comparatively new concept for employees in India (PTI, 2022), and hence, this scale can identify the impact of the varying degrees of time autonomy on their wellbeing, motivation, and many other outputs leading to management considering the need to include or exclude the specific autonomy in their human resource practices (HRP).

The next factor in AHWS was scheduling autonomy. Work scheduling autonomy pertains to the level of discretion employees possess regarding when to carry out specific tasks, including scheduling and sequencing (Breaugh, 1985). It is important to note that work scheduling autonomy differs from flexitime autonomy, as the latter specifically relates to employees’ autonomy in determining the start and end times of their tasks rather than the order in which tasks are performed (Spiegelaere et al., 2016).

**Fig. 1:**
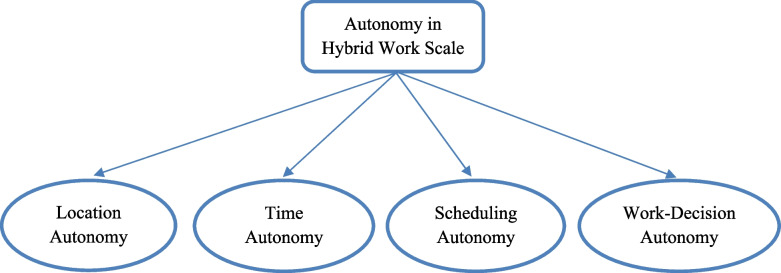
Dimensions of the variable “autonomy in hybrid work”

Work decision autonomy was the last factor of the AHWS. It refers to the extent to which individuals have the authority and discretion to make choices and judgments about their work-related tasks, responsibilities, and processes (Morgeson & Humphrey, 2006). While earlier scales, such as those by Morgeson and Humphrey (2006), provided valuable insights by employing general items like “the job allows me to make my own decisions about how to schedule my work” and “the job provides me with significant autonomy in making decisions” to measure scheduling and decision-making autonomy in traditional workplace settings, our current study takes a more nuanced approach. We present in-depth and specific questions such as “I can decide the timing and duration of my breaks” and “I can decide to utilize tools and software applications of my choice to work on tasks,” aiming to capture the intricacies of autonomy in this evolving work landscape. The four dimensions of AHWS-18 (see Fig. 1) demonstrated robust psychometric properties, including good reliability, test–retest correlation, convergent/discriminant validity, and factor loading.

Cronbach alpha coefficients for both the AHWS scale and its subfactors exceeded the recommended threshold of 0.70, with a particularly meaningful coefficient of 0.825 in this study. The identified factors align with the theoretical construct of autonomy in the contemporary world, as outlined by Korunka & Kubicek’s, (2017). Therefore, the study’s findings define autonomy in hybrid work as the employees’ discretion over their work location, work time, work scheduling, and work decision-making while functioning in a combination of remote and in-office modes.

## Conclusion and implications of the study

According to estimates from the World Health Organization (WHO), approximately 15% of working-age adults in India have experienced a mental disorder. Notably, workplace-related stress has emerged as the primary factor influencing the mental health of employees. A survey conducted by Deloitte found that a significant 47% of professionals report experiencing stress directly attributable to their workplace (Fisher et al., 2023). Also, a Gallup workplace report states that employee stress is at an all-time high (Beheshti, 2022). With the high job demands leading to stress in IT organizations in India, it is an immense challenge for the management to retain and furnish job satisfaction to its employees. From existing theoretical and empirical evidence, we comprehend that autonomy plays a huge role in buffering the effects of high job demands on employee stress. However, with the emergence of a hybrid work environment post-COVID-19 in India, the existing scales in the literature lack various factors to measure the level of autonomy in this contemporary workplace.

Top management, managers, and HR specialists of IT companies can utilize the scale to measure the effectiveness of the diverse autonomy provided to the employees while working in a hybrid model on their stress, wellbeing, productivity, and other employee outcomes. The results from utilizing the scale can guide human resource management (HRM) policymakers in creating a suitable work environment for their employees. In addition, the four dimensions uncovered from the analysis create a foundation of the autonomy at hybrid work concept that can generate input for HRP in organizations adopting the new approach to the workplace.

Additionally, the study contributes to the proficiency of existing literature and theories on autonomy in work. The developed scale can be used to extend theories like self-determination, job characteristics, and job demand-control model in the context of a hybrid work environment. The theoretical resonance of this scale echoes the evolving landscape of the contemporary workplace, underscoring the significance of autonomy as a multifaceted construct with implications extending beyond conventional office settings.

## Data Availability

The datasets generated and/or analyzed during the current study are not publicly available due to the confidentiality clause but are available from the corresponding author upon reasonable request.

## Abbreviations

AHWS: Autonomy in Hybrid Work Scale
IT: Information technology
EFA: Exploratory Factor Analysis
CFA: Confirmatory Factor Analysis
CR: Composite reliability
AVE: Average variance extracted
HTMT: Heterotrait-monotrait
WLA: Work location autonomy
WTA: Work time autonomy
WSA: Work schedule autonomy
WDA: Work decision autonomy
CMIN/df: Discrepancy divided by degree of freedom
RMSEA: Root mean square error of approximation
GFI: Goodness-of-Fit Index
AGFI: Adjusted Goodness of Fit Index
NFI: Normed Fit Index
CFI: Comparative Fit Index
TLI: Tucker Lewis index
HRM: Human resource management
HRP: Human resource practices
WHO: World Health Organization
